# Automated Assessment of Pelvic Longitudinal Rotation Using Computer Vision in Canine Hip Dysplasia Screening

**DOI:** 10.3390/vetsci11120630

**Published:** 2024-12-07

**Authors:** Pedro Franco-Gonçalo, Pedro Leite, Sofia Alves-Pimenta, Bruno Colaço, Lio Gonçalves, Vítor Filipe, Fintan McEvoy, Manuel Ferreira, Mário Ginja

**Affiliations:** 1 Department of Veterinary Science, University of Trás-os-Montes and Alto Douro (UTAD), 5000-801 Vila Real, Portugal; pedrofranco@utad.pt; 2 Veterinary and Animal Science Research Centre (CECAV), University of Trás-os-Montes and Alto Douro (UTAD), 5000-801 Vila Real, Portugal; salves@utad.pt (S.A.-P.); bcolaco@utad.pt (B.C.); 3 Associate Laboratory for Animal and Veterinary Sciences (AL4AnimalS), 5000-801 Vila Real, Portugal; 4 Neadvance Machine Vision SA, 4705-002 Braga, Portugal; pleite@neadvance.com (P.L.);; 5 Department of Animal Science, University of Trás-os-Montes and Alto Douro (UTAD), 5000-801 Vila Real, Portugal; 6 School of Science and Technology, University of Trás-os-Montes and Alto Douro (UTAD), 5000-801 Vila Real, Portugal; lgoncalv@utad.pt (L.G.); vfilipe@utad.pt (V.F.); 7 Department of Engineering, University of Trás-os-Montes and Alto Douro (UTAD), 5000-801 Vila Real, Portugal; 8 Institute for Systems and Computer Engineering (INESC-TEC), Technology and Science, 4200-465 Porto, Portugal; 9 Department of Veterinary Clinical Sciences, Faculty of Health and Medical Sciences, University of Copenhagen, 1165 Copenhagen, Denmark

**Keywords:** canine hip dysplasia, pelvic rotation, computer vision model, asymmetry in obturator foramina areas

## Abstract

Canine hip dysplasia (CHD) screening relies on accurate positioning in the ventrodorsal hip extended (VDHE) view, as even mild pelvic rotation can affect CHD scoring and impact breeding decisions. This study aimed to assess the association between pelvic rotation and asymmetry in obturator foramina areas (AOFAs) and to develop a computer vision model for automated AOFA measurement. In the first part, 203 radiographs were analyzed to examine the relationship between pelvic rotation, assessed through asymmetry in iliac wing and obturator foramina widths (AOFWs), and AOFAs. A significant association was found between pelvic rotation and AOFA, with AOFW showing a stronger correlation (*R*^2^ = 0.92, *p* < 0.01). AOFW rotation values were categorized into minimal (n = 71), moderate (n = 41), marked (n = 37), and extreme (n = 54) groups, corresponding to mean AOFA ± standard deviation values of 33.28 ± 27.25, 54.73 ± 27.98, 85.85 ± 41.31, and 160.68 ± 64.20 mm^2^, respectively. ANOVA and post hoc testing confirmed significant differences in AOFA across these groups (*p* < 0.01). In part two, the dataset was expanded to 312 images to develop the automated AOFA model, with 80% allocated for training, 10% for validation, and 10% for testing. On the 32 test images, the model achieved high segmentation accuracy (Dice score = 0.96; Intersection over Union = 0.93), closely aligning with examiner measurements. Paired *t*-tests indicated no significant differences between the examiner and model’s outputs (*p* > 0.05), though the Bland–Altman analysis identified occasional discrepancies. The model demonstrated excellent reliability (ICC = 0.99) with a standard error of 17.18 mm^2^. A threshold of 50.46 mm^2^ enabled effective differentiation between acceptable and excessive pelvic rotation. With additional training data, further improvements in precision are expected, enhancing the model’s clinical utility.

## Simple Summary

Canine hip dysplasia is a painful condition common in large dog breeds, leading to joint instability and osteoarthritis. Accurate diagnosis is crucial for guiding breeding decisions to help reduce its prevalence. However, evaluating hip health status can be challenging, as minor positioning changes during X-rays can distort images and hinder proper hip joint assessment. In this study, we developed an artificial intelligence tool for the automatic evaluation of pelvic alignment in X-rays. By detecting subtle asymmetries in bone structure, the tool determines whether a dog’s hips are properly aligned. Our findings showed that the artificial intelligence tool performed as accurately as an expert human examiner in detecting misalignment. This automated approach could improve the reliability of canine hip dysplasia screenings, saving veterinarians time and reducing misdiagnosis risks due to human error. Ultimately, this technology has the potential to enhance medical care for dogs and support breeders in making more informed choices, contributing to canine health improvements and reducing canine hip dysplasia incidence in future generations.

## 1. Introduction

Canine hip dysplasia (CHD) is a polygenic, non-congenital orthopedic disorder that predominantly affects large and giant dog breeds. It is initially characterized by hip instability and a lack of congruency between the femoral head and the acetabulum, which eventually leads to secondary osteoarthritis, lameness, and physical disability [1]. Although diagnostic genetic tests have been developed, they have yet to reach sufficient diagnostic accuracy [2,3]. Consequently, phenotypic assessment, by means of radiographic examination, remains the cornerstone of diagnosis and screening. This approach is crucial for informing selective breeding practices, thereby helping to prevent the transmission of defective genes to offspring [1].

The ventrodorsal hip extended (VDHE) view, introduced in the 1960s, has since become the standard radiographic technique, widely adopted by screening organizations worldwide for CHD certification. To ensure high-quality radiographs, specific guidelines were established, requiring the dog to be positioned in dorsal recumbency on the X-ray table with hind limbs extended caudally and the femurs slightly internally rotated, ensuring a symmetric pelvis, parallel and fully extended femurs, and patellas centered within the femoral trochlea [4].

In human medicine, diagnosing developmental dysplasia of the hip and conducting follow-up assessments typically involves measuring the acetabular index [5,6]. However, changes in pelvic positioning and rotation can result in variations in acetabular index measurements, which may affect diagnostic accuracy and influence decisions regarding surgical treatment [5,6]. Similarly, in CHD radiographic assessment, even slight pelvic tilting to the right or left distorts the projection of the femoral head and acetabulum, leading to altered overlap on the upper and lower sides and potentially resulting in diagnostic inaccuracies [7,8,9].

In veterinary medicine, screening organizations such as the Fédération Cynologique Internationale, the Orthopedic Foundation for Animals, and the British Veterinary Association/Kennel Club utilize common evaluation parameters such as the Norberg Angle (NA) and visual assessment of hip subluxation/congruency to classify CHD in the VDHE view [1,10].

These metrics are especially critical in identifying normal joints or early-stage cases without evidence of osteoarthritic changes [10]. However, inappropriate positioning of the dog during X-ray acquisition can distort the anatomical relationship between the femoral head and acetabulum, compromising radiographic positioning quality. The displacement of critical landmarks, such as the cranial acetabular rim, is essential for accurate CHD scoring [11]. Misinterpretation of these landmarks by veterinarians may result in the selection of improper breeding candidates or the unwarranted exclusion of suitable dogs [11]. This underscores the importance of conducting a thorough evaluation of positioning quality before moving forward with the appraisal of hip joint status.

Previous studies explored and quantified the impact of pelvic tilting along the long axis, femoral pronation and supination, and femoral abduction and adduction on the NA and hip joint congruency assessment in VDHE views [7,8,9,12,13]. Both clinical and cadaveric studies showed that pelvic tilting along the long axis of the body benefits the perceived radiographic hip joint relationship, indicated by the NA, on the topside of the rotation while impairing it on the underside [7,9]. Similarly, femoral pronation was found to enhance the apparent radiographic proximity between the femoral head and the acetabulum, whereas femoral supination diminishes it [12]. In addition, a recent study provided evidence that femoral abduction overvalues both the NA and the hip congruency index, while femoral adduction undervalues these parameters [13].

Currently, radiographic image positioning quality is assessed using standard Digital Imaging and Communications in Medicine (DICOM) viewing software, requiring veterinarians to manually measure distances and angles. This process is labor-intensive and time-consuming, often leading clinicians, in particular, to rely on subjective judgment based on their expertise to decide whether to accept or reject radiographs that are not perfectly positioned [11]. Simultaneously, they empirically assess how specific positioning errors affect measurements, making approximate adjustments that are not always precise [11].

Given these challenges, there is an increasing demand for automated software that can quickly and accurately evaluate radiographic positioning quality. With advances in computer vision and artificial intelligence (AI), the medical diagnostic industry is evolving, with new tools automating these tasks to streamline the process and improve efficiency [14]. These technologies often rely on annotated images as training data, allowing them to learn how to identify key anatomical landmarks and accurately evaluate new images post-training, offering a more objective and reliable approach to radiographic assessments [14].

Pelvic rotation along the long axis of the body is widely acknowledged as a major factor of inaccurate assessments of hip joint status, contributing to a low intra-rater agreement [11], which raises concerns about the reliability of the VDHE view in effectively evaluating CHD [15,16,17,18]. Recognizing that pelvic rotation can be subjectively identified through the asymmetrical projection of its two halves, researchers sought to objectively quantify pelvic rotation by examining anatomical pelvic asymmetry. Their findings demonstrated a strong correlation between the degree of rotation and the asymmetry observed in the widths of the iliac wings and the maximum widths of the obturator foramina [7].

The primary objectives of this research were to investigate the association between the degree of pelvic rotation—calculated separately using the asymmetry in iliac wings widths (AIWWs) and obturator foramina widths (AOFWs)—and the asymmetry in obturator foramina areas (AOFAs) and to determine the reliability and agreement between an experienced examiner (P.F.-G.) and an automated Dys4Vet AOFA measurement model. Our null hypotheses were that the AOFA means do not differ across various pelvic rotation degrees calculated using the AOFW and that there is no significant difference between the AOFA means determined by the examiner and the automated software.

## 2. Materials and Methods

This was a retrospective study based on the evaluation of 312 VDHE radiographs from dogs, performed between 2010 and 2024, for CHD screening. The radiographs were sourced from the Veterinary Teaching Hospital of the University of Trás-os-Montes and Alto Douro and the Danish Kennel Club databases. The data recorded included age, breed, and sex.

The inclusion criteria required that dogs be over 12 months of age. Although some radiographs lacked specific age information, they were validated based on skeletal maturity, with the understanding that only dogs over 12 months old are eligible for CHD screening. Radiographs also needed to be properly collimated, including both the iliac crests and patellae. Additionally, they must not exhibit visible pathological lesions, damage, or pelvic deformities that could obscure or affect the identification of critical radiographic landmarks, such as the ilium and the obturator foramina. Due to variability in the quality of the radiographs collected from the databases, not all images met the criteria for CHD screening. Consequently, in the first part of the study, each dog contributed between two and four radiographs. In the second part of the study, the dataset was expanded to include additional images, each representing a different dog. Given the retrospective and observational nature of the study, ethical committee approval and owner consent were waived.

### 2.1. Study Design

This study was conducted in two parts. In the first part, a total of 203 radiographs from the database were analyzed, with each dog contributing between two to four repeated views, each exhibiting varying degrees of pelvic rotation, to investigate the relationship between pelvic longitudinal rotation, calculated separately using AIWW and AOFW, and AOFA. This sample size was chosen to ensure sufficient statistical power to detect a statistically significant effect of pelvic rotation on AOFA in an ANOVA test, substantially exceeding the minimum calculated sample size of 180, determined a priori via power analysis using G*Power software. The power analysis was conducted with an alpha level of 0.05, an assumed effect size of 0.25, and a statistical power threshold of 0.80 [19]. By exceeding the calculated sample size, we established a sufficiently robust sample to support the subsequent examination of AOFA across varying degrees of pelvic rotation. Pelvic rotation values were converted to positive degrees and categorized into four rotation groups: minimal (0° to 2°), moderate (3° to 4°), marked (5° to 6°), and extreme (≥7°). Values falling between two groups were rounded to the nearest integer. Mean AOFA values were then compared across rotation groups to evaluate potential differences.

In the second part of the study, additional radiographs were randomly selected from the database to expand the dataset, resulting in a total of 312 images. This complete dataset was then randomly divided into three groups for the development of the Dys4Vet AOFA model: 80% (248 images) for training, 10% (32 images) for validation, and 10% (32 images) for testing. The 32 radiographs in the testing group were used to compare the manual measurements taken by the examiner with the model’s outputs.

### 2.2. Radiographic Measurements

**Figure 1:**
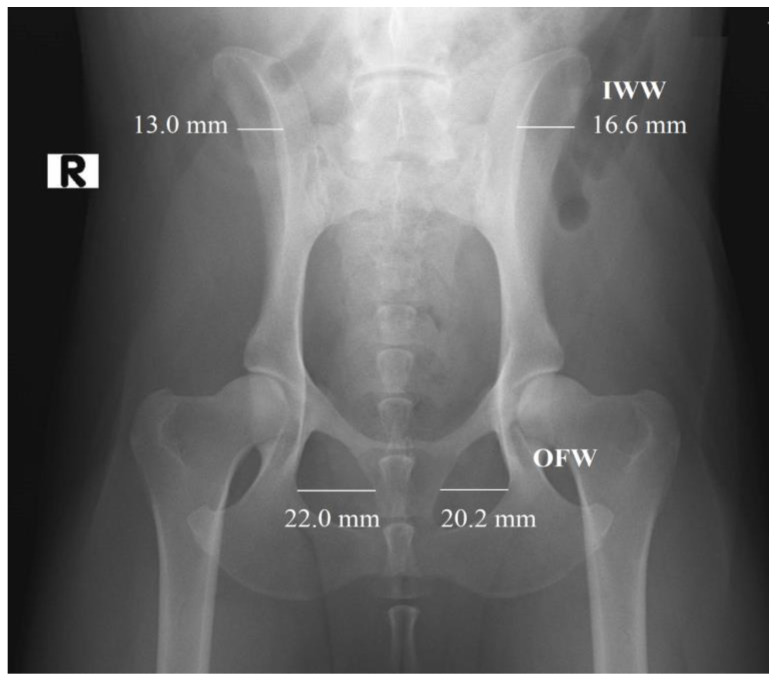
Measurement of the widths of the right and left iliac wings (IWW) in millimeters (mm): horizontal lines were drawn between the dorsal and ventral iliac cortices at the cranial aspect of the sacroiliac joint on both sides. Measurement of the widths of the right and left obturator foramina (OFW) in mm: horizontal lines were drawn between the medial and lateral aspects of each foramen at their widest points. Asymmetry was calculated as the difference between the two measurements (largest minus smallest). R indicates the right side.

**Figure 2:**
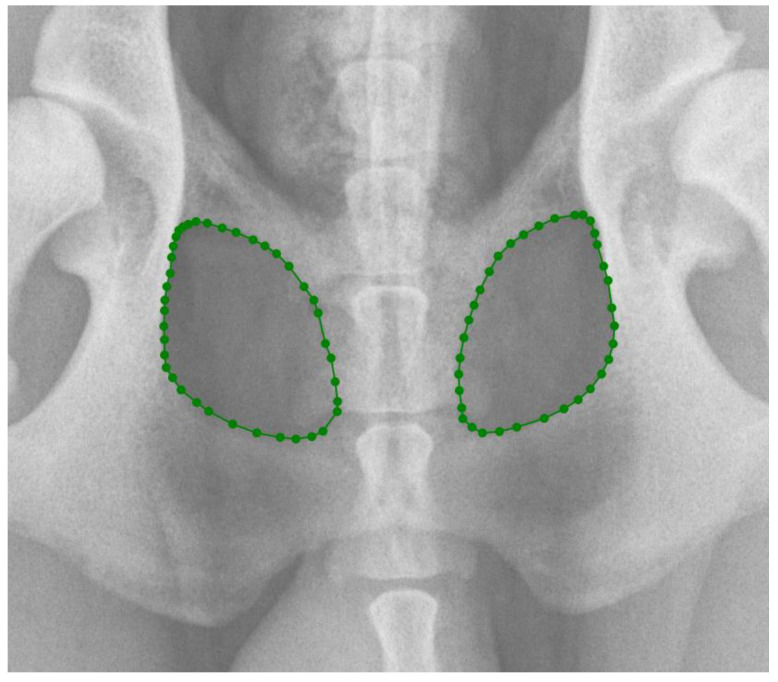
Delimitation of the obturator foramina using the LabelMe annotation tool for the calculation of the asymmetry in obturator foramina areas (AOFAs) in ventrodorsal hip extended (VDHE) view. The obturator foramina areas are delimitated by greens points.

In the first part of the study, 203 radiographs in DICOM format were manually annotated using the polygonal image annotation tool, LabelMe [20], to delineate the right and left obturator foramina. The AIWW was calculated by measuring the widths of the right and left iliac wings in millimeters. A horizontal line was drawn between the dorsal and ventral iliac cortices at the cranial aspect of the sacroiliac joint on both sides (Figure 1). The AIWW was determined by subtracting the width of the right wing from that of the left [10]. The AOFW was calculated by measuring the widths of the right and left obturator foramina in millimeters. A horizontal line was drawn between the medial and lateral aspects of each foramen at its widest points (Figure 1). The AOFW was determined by subtracting the width of the right obturator foramen from that of the left [10]. The AIWW and AOFW, measured in millimeters (*x*), were used separately to estimate the degrees of pelvic rotation (*y*) through the following regression equations: y = 0.997x + 0.061 for AIWW and y = 1.644x − 0.912 for AOFW [10]. The AOFA was calculated by measuring the areas of the right and left obturator foramina in square millimeters. For this purpose, the areas were outlined using semantic segmentation, employing pixel-wise labeling to trace the boundaries of both obturator foramina (Figure 2). The AOFA was determined by subtracting the area of the right obturator foramen from that of the left.

In the second part, the examiner performed AOFA measurements on an additional 109 radiographs, resulting in a total of 312 annotated images. Following training, the Dys4Vet model generated AOFA calculations on the 32 images designated for testing.

### 2.3. Model Overview

The objective of this model was to automatically calculate the AOFA by accurately segmenting both obturator foramina from VDHE radiographic images. Data preparation for model development involved manually outlining the boundaries of both obturator foramina in 280 randomly selected DICOM images from the original set of 312, which were used for training and validation, using the LabelMe annotation tool [20]. All measurements were carried out by P.F.-G., serving as the reference for precise area labeling. Afterward, the images were converted to PNG format and resized to 448 × 448 pixels. These annotated images, referred to as ground truth data, were used to train and validate the model to recognize the obturator foramina in novel images. The number of images was chosen arbitrarily and would have been increased had the model’s performance been unsatisfactory.

### 2.4. Model Architecture

U-Net was selected as the primary architecture for the semantic segmentation task of identifying and measuring the obturator foramina in radiographs [21]. U-Net has demonstrated high efficacy in medical image segmentation, making it widely applicable in similar contexts [22]. U-Net consists of two primary components: an encoder, responsible for extracting critical features from the image, and a decoder, which applies these features to generate a “segmentation mask” delineating the obturator foramina. For optimal feature extraction within the U-Net framework, EfficientNet-B5 was implemented as the encoder due to its great accuracy and efficient computational power. This model configuration enabled accurate and efficient segmentation of the target regions.

### 2.5. Model Training

The model was trained using the Adam optimizer algorithm, which fine-tunes the learning process by adjusting its updates at each training step. A learning rate of 0.001 was selected to ensure steady improvements over time. A batch size of 8 was used, meaning the model processed 8 images at a time, allowing it to focus more closely on the details within each set of images.

To make the model more robust, data augmentation techniques were applied during training. These techniques included flipping, rotating, and adjusting the contrast and brightness of the images, which introduced variety to the data and helped prevent overfitting, by ensuring the model did not become overly focused on specific patterns in the training set. After each learning cycle, these augmentation techniques were applied to the images with a probability of happening set at 0.5, ensuring that the training images varied continuously, further enhancing the model’s adaptability.

Training was conducted on an NVIDIA RTX 2000 Ada system with 8 GB of VRAM, allowing for faster processing. The experiments were carried out using Keras with TensorFlow, supported by CUDA 11.2 to leverage GPU capabilities for improved performance.

### 2.6. Model Segmentation Performance

The model’s segmentation performance was evaluated using the 32 images reserved for testing. Two key metrics were used to assess its performance: the Dice score (DS) and Intersection over Union (IOU). The DS metric evaluates the similarity between two sets of images, while the IOU measures the degree of overlap between the predicted segmentation masks and the ground truth [23].

### 2.7. Statistical Analysis

Statistical analysis was performed using the SPSS software (SPSS Statistics for Windows 128 Version 27.0: IBM Corp., Armonk, NY, USA). A *p*-value of <0.05 was considered statistically significant across the entire study. Hedge’s *g* was used to measure effect size: small ≥ 0.20, medium ≥ 0.50, large ≥ 0.80, and very large ≥ 1 [24,25,26]. The achieved power was calculated post hoc using the G*Power software (version 3.1.9.7) [19]. The Central Limit Theorem was adopted, which stipulates that for sufficiently large sample sizes (*n* > 30), the distribution tends to be normally distributed, regardless of the original distribution of the variable in the population, and so, parametric test for data analysis were used [27].

In the first part, Pearson correlation analyses were used to determine the association between the degrees of pelvic rotation—calculated separately using the AIWW and the AOFW—and the AOFA. The homogeneity of variance was checked, and the Welch’s ANOVA followed by the post hoc Games–Howell test was used to compare mean AOFA values between categories of pelvic rotation calculated using the AOFW.

In the second part, the paired *t*-test was used to determine if there was a systematic difference between the examiner and the Dys4Vet model [28]. A Bland–Altman analysis was conducted to assess the pattern and extent of agreement between the two measurement methods. The 95% limits of agreement (LAs) were calculated as the mean differences ± 1.96 standard deviation (SD). When the 95% confidence interval (CI) of the mean differences includes zero, measurements are considered to be in agreement, and when the 95% lower and upper LA are small, measurements are considered to be equivalent [29,30]. The intraclass correlation coefficient (ICC_3,1_ absolute agreement model) was used to evaluate the inter-rater reliability between the two measurement methods, alongside the standard error of measurement (SEM) to assess the precision of individual measurements [28,31,32]. ICC values were interpreted as follows: random (0), poor (<0.5), moderate (0.5–0.75), good (0.75–0.9), excellent (>0.9), and perfect (1) reliability [31]. A lower limit 95% CI of ICC > 0.75 was defined as adequate reliability [31,33].

## 3. Results

The 312 images included in this study belonged to 199 dogs, predominantly from five main breeds: German Shepherds (32 dogs, 18.39%), Labrador Retrievers (29 dogs, 16.67%), Estrela Mountain Dogs (23 dogs, 13.22%), Portuguese Pointers (19 dogs, 10.92%), and French Mastiffs (17 dogs, 7.78%), along with several other breeds. There were 46% males and 54% females.

### 3.1. First Part: Association Between Pelvic Rotation and Asymmetry of Obturator Foramina Areas

The AOFA was significantly correlated with the degrees of pelvic rotation calculated using the AIWW (*r* = 0.76; 95% CI [0.7, 0.82], *p* < 0.001, power = 1). A significant linear regression coefficient was observed (*R*^2^ = 0.58, *p* < 0.001).

The AOFA was significantly correlated with the degrees of pelvic rotation calculated using the AOFW (*r* = 0.96; 95% CI [0.94, 0.97], *p* < 0.001, power = 1). A significant linear regression coefficient was observed (*R*^2^ = 0.92, *p* < 0.001).

**Figure 3:**
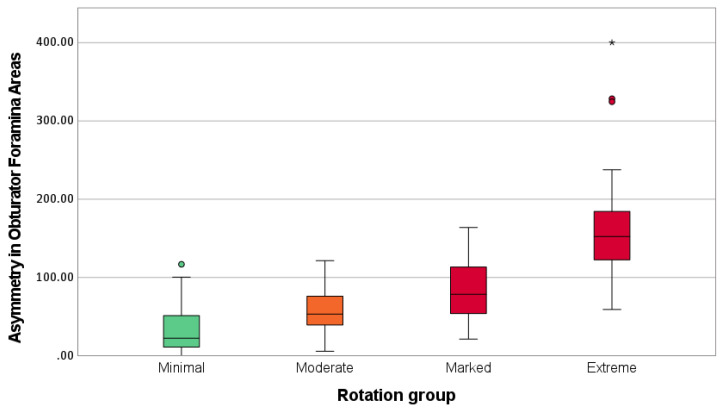
Box-and-Whisker plot presenting the asymmetry in obturator foramina areas (mm^2^) categorized by pelvic rotation group (minimal to extreme). Green and red dots represent outliers falling outside the whiskers (values > 1.5 times the interquartile range). Asterisk (*) marks an extreme outlier (value > 3 times the interquartile range).

With AOFW serving as the reference, a total of 71 out of 203 (35%) pelvic rotation values were included in the minimal rotation group, with an AOFA mean ± SD of 33.28 ± 27.25 mm^2^. In the moderate rotation group, 41 out of 203 (20%) values were included, with an AOFA mean ± SD of 54.73 ± 27.98 mm^2^. In the marked rotation group, 37 out of 203 (18%) values were included, with an AOFA mean ± SD of 85.85 ± 41.31 mm^2^. Finally, in the extreme rotation group, 54 out of 203 (27%) values were included, with an AOFA mean ± SD of 160.68 ± 64.20 mm^2^. The Levene’s test indicated unequal variances among the categories (*p* < 0.001). The Welch’s ANOVA followed by the post hoc Games–Howell test indicated statistically significant differences between all the groups (*p* < 0.01) (Table 1; Figure 3). Pairwise comparisons yielded the following effect sizes and corresponding power values: *g*_minimal-moderate_ = 0.78 (power = 0.72), *g*_minimal-marked_ = 1.5 (power = 0.99), *g*_minimal-extreme_ = 2.58 (power = 1), *g*_moderate-marked_ = 0.88 (power = 0.75), *g*_moderate-extreme_ = 2.14 (power = 1), and *g*_marked-extreme_ = 1.39 (power = 0.99).

### 3.2. Second Part: Comparison of Dys4Vet AOFA Model Output with Examiner’s Measurements and Segmentation Performance

**Figure 4:**
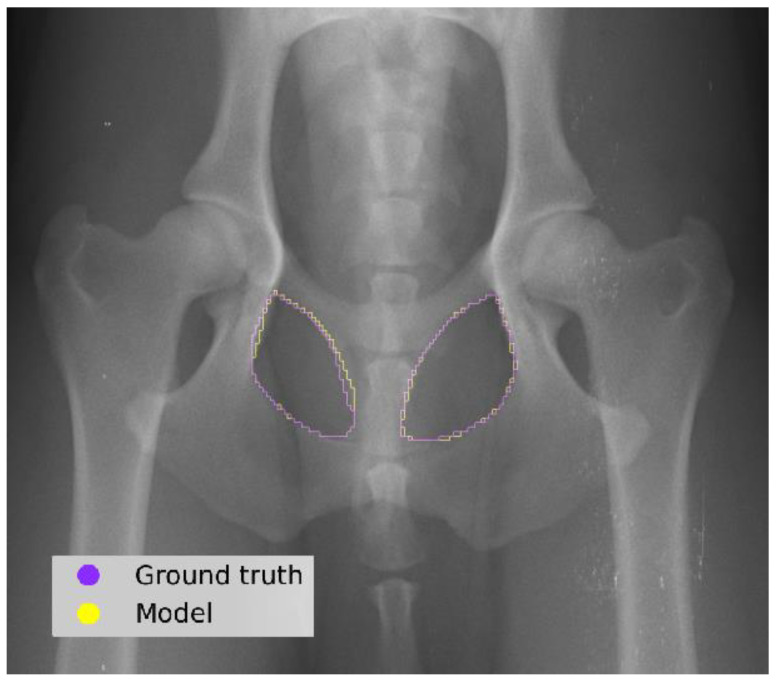
Comparison between the ground truth (examiner) and the predictions made by the model in a test image.

The model segmentation performance achieved a DS of 0.96 and an IOU score of 0.93 (Figure 4).

**Figure 5:**
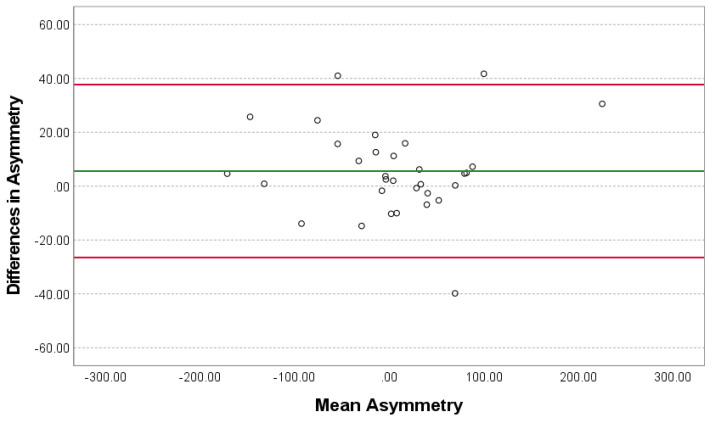
Differences in asymmetry in obturator foramina areas (mm^2^) between the model and the examiner in the 32-image test subset. The green line represents the mean of the differences (5.59), and the red lines represent the lower and upper 95% limits of agreement, −26.51 and 37.69, respectively.

The AOFA mean ± SD for the model was 7.41 ± 78.90 mm^2^, and for the examiner, was 1.82 ± 78.90 mm^2^. The AOFA mean difference ± SD between the model and the examiner was 5.59 ± 16.38 (95% CI [−0.32, 11.50], *p* = 0.06, *g* = 0.34, power = 0.5, in paired *t*-test) (Figure 5). The ICC for AOFA between the model and the examiner was 0.99 (95% CI [0.98, 0.99], *p* < 0.001), with a SEM of 17.18 mm^2^.

## 4. Discussion

Proper positioning and optimal exposure technique are critical for producing radiographs of sufficient diagnostic quality to accurately score CHD [9]. In a VDHE view, the dog’s dorsal plane should be parallel and close to the X-ray table, with the body and limbs fully extended and aligned. The X-ray beam must be centered on the pelvis and aligned with the dog’s longitudinal axis. Any tilting of the pelvis to the right or left will alter the radiographic anatomical projection of the pelvis. When the pelvis rotates to the dog’s right side, the anatomical structures on the left half of the pelvis (the topside) become more concealed and angled, while those on the left (the underside) are more exposed, allowing the ventral plane portion of that half to be fully visible. The structures on the topside also undergo geometric magnification, as they are closer to the X-ray tube, causing them to appear slightly enlarged on the radiograph. To address these phenomena, previous studies have successfully used the approach of comparing asymmetries between the two pelvic halves to assess the quality of radiographic positioning in VDHE views [12,34]. In this study, we tried to refine and apply the AOFA methodology to a computer vision measurement model.

The findings of this study provide evidence for rejecting the first null hypothesis, as the AOFA was found to vary significantly across different degrees of pelvic rotation calculated using the AOFW (*p* < 0.05 in ANOVA and post hoc comparisons). Conversely, we fail to reject the second null hypothesis, as there was no statistically significant difference between the AOFA determined by the examiner and those measured by the Dys4Vet AOFA model (*p* > 0.05 in the paired *t*-test).

To evaluate the strength of the relationship between pelvic rotation and the AOFA, a Pearson correlation was applied. The analysis demonstrated a strong positive correlation between pelvic rotation degrees, calculated separately using AIWW and AOFW, and the AOFA. The *R*^2^ for pelvic rotation using AIWW shows that 58% of the variance in pelvic rotation is explained by AOFA (*p* < 0.001), while the *R*^2^ for AOFW indicates that 92% of the variance is explained by AOFA (*p* < 0.001). We chose the AOFW method over AIWW to calculate pelvic rotation degrees for the association with AOFA, primarily due to the significantly higher *R*^2^ value for AOFW. This stronger correlation is expected, as AOFW is closely linked to AOFA; the maximum width of the obturator foramen is a crucial element in the calculation of its area (i.e., *A* = π*a*b, where *a* is the longest radius and *b* the shortest), since the obturator foramen is elliptical in shape. The AOFA has already been shown to be strongly correlated with pelvic rotation (*R* = 1.0) in a previous cadaveric study [10]. However, it was not recommended as a predictor of pelvic rotation due to the absence of statistically significant differences in mean asymmetry across the studied degrees of rotation [10]. This lack of significance could be attributed to the small sample size used in the study, which limited its statistical power and reliability, among other possible factors.

Another key reason for selecting the AOFW method is the inherent variability in ilium bone morphology across individuals, which contrasts with the relative consistency of the obturator foramina. In computer vision models, it is generally more effective to focus on features that exhibit less variability and greater consistency, as this leads to improved model training and, ultimately, better performance. It is known that morphological diversity is a sign of adaptation to challenging environments, and individuals under stress often develop various forms of asymmetry [35,36,37]. Previous studies have shown that the pelvic canal and the ilium are among the most variable regions of the pelvis in different animal species, including dogs, due to their roles in locomotion, muscle attachment, and weight-bearing functions [38,39,40]. A recent geometric morphometry study in dogs demonstrated that these structures are shaped by breed-specific characteristics. For example, Gundogs (Retrievers and Spaniels) exhibited a narrower pelvic canal and a broader *crista iliaca*, while Terriers had a wider pelvic canal and a smaller *crista iliaca* [40]. This morphological difference was also noticeable between sexes; males tended to have a narrower pelvic canal and a higher *crista iliaca* compared to females [40,41]. Additionally, male pelvises displayed more significant directional asymmetries (consistent differences between the right and left sides of the pelvis), with the right side being more prominent, particularly in the *crista iliaca* and *arcus ischiadicus* [40]. This suggests that areas of the ilium, especially the wings, are more prone to shape variation, which can introduce errors and discrepancies in the analysis of a population sample. In contrast, the shape of the obturator foramen is highly constrained due to its primary function as a passageway for neurovascular structures and is less influenced by biomechanical forces [42]. Therefore, it is reasonable to infer that the obturator foramina exhibit less morphological variability across individuals, breeds, and sexes. In our opinion, the obturator foramina provide a more reliable and stable anatomical feature for analyzing radiographic positional asymmetry, making them a more suitable parameter for developing a computer vision model compared to the more variable ilium wings. Furthermore, the sharp contrast between the radiolucent orifice and its radiopaque contour in the obturator foramina offers clear, well-defined grayscale intensity patterns, which can be easily segmented and detected by AI algorithms. This distinct anatomical feature improves model training and enhances the accuracy of detection in radiographic images.

Welch’s ANOVA of AOFA values in the pelvic rotation degree groups revealed a statistically significant main effect (*p* < 0.001), indicating that not all degree groups had the same mean AOFA value. Post hoc comparisons using the Games–Howell post hoc procedure were conducted to determine which pairs of the four groups’ means differed significantly. The results show a gradual increase in mean AOFA values with the degree of pelvic rotation across the different groups. The minimal group had a significantly lower mean AOFA value compared to the moderate, marked, and extreme groups (*p* < 0.001). Similarly, the moderate group had a significantly lower mean AOFA value than the marked and extreme groups (*p* < 0.01), and the marked group had a significantly lower mean AOFA value than the extreme group (*p* < 0.001). Furthermore, pairwise comparisons yielded effect sizes and power values that validate the significance of these differences. Hedges’ *g* values ranged from medium to very large (0.78 to 2.58), indicating substantial differences in AOFA between the pelvic rotation groups. The corresponding power values, mostly close to or above 0.80, confirm that the study design was robust and provided sufficient power to detect these differences with a high level of confidence. These robust effect sizes and high power values demonstrate that the observed differences in AOFA are not only statistically significant but also of practical relevance. Therefore, the null hypothesis is rejected, which supports the assumption that the AOFA can help predict different ranges of pelvic rotation, assisting in the evaluation of radiographic positioning quality and aiding to determine the suitability of radiographs for CHD evaluation.

Our automated AOFA measurement model demonstrated high accuracy in segmenting the obturator foramina regions in the test set, with a strong similarity and overlap with the ground truth masks. When compared to other AI-based radiographic segmentation models, such as those developed by Rouzrokh et al. for measuring acetabular component angles in total hip arthroplasty, which focus on surgical outcomes and implant positioning, our model showed superior segmentation accuracy [43]. Their study reported a DS of 0.90 for acetabular component segmentation in the anteversion angle model, the highest among their tested models [43]. In contrast, our model achieved a DS of 0.96 for obturator foramina segmentation, emphasizing its precision in delineating complex pelvic structures. Similarly, when compared with the segmentation models developed by Moreira da Silva et al. for femur and acetabulum segmentation aimed at quantifying hip joint congruency for CHD diagnosis, our model exhibited comparable performance. Their study employed a U-Net-based segmentation model, achieving a DS of 0.98 for femur segmentation and 0.93 for acetabulum segmentation [44]. Our model’s superior or comparable performance to existing AI-based segmentation systems demonstrates its potential to enhance diagnostic precision and streamline workflows in both veterinary and human medicine.

To assess its suitability for clinical practice, we compared the model’s performance with that of an experienced examiner. The results demonstrated acceptable reliability and agreement between the two measurement methods. The paired *t*-test (*p* > 0.05) indicates that the mean difference observed (5.59 mm^2^) is not statistically significant. This suggests that we fail to reject the null hypothesis, implying there is no strong evidence to claim that the two methods yield significantly different results. However, the Bland–Altman plot showed that the 95% LAs, ranging from −26.51 to 37.69 mm^2^, are somewhat wide, suggesting occasional larger discrepancies between the model and examiner measurements. While this indicates room for improvement in terms of precision, the majority of the differences fall within an acceptable range, with only a few notable outliers. Nevertheless, while the current sample’s statistical power (0.5) introduces some limitations in confidently concluding that the two methods yield comparable results, it still provides valuable preliminary insights. Given the small effect size (0.34), a sample size of approximately 70 dogs would enhance statistical power to 0.80, supporting a more reliable determination of any potential differences, or lack thereof, between the methods. While it was possible to increase the study sample, this would require reallocating images from the training dataset to the model’s testing and validation process, which would almost certainly degrade the model’s overall performance. In another instance, the ICC was 0.99, with CI ranging from 0.98 to 0.99, reflecting excellent reliability between the two measurement methods. However, the model’s precision is suboptimal, with a SEM of 17.18 mm^2^, indicating deviations from the examiner’s values by around ±17.18 mm^2^ in 68% of cases, emphasizing the need for improved precision.

One limitation of the study is that we compared the model to a single examiner, without assessing agreement and reliability across multiple examiners. Importantly, this provides some confidence in the results, as the model’s tendency to overestimate measurements could be attributed to individual variability from the examiner, rather than a fundamental flaw in the model itself. Further validation with additional examiners could confirm that the model’s accuracy is consistent across different assessments. Another limitation may be that the craniocaudal inclination of the pelvis was not monitored, and previous studies have demonstrated that it influences the imaging projection of pelvic structures [45]. A notable strength of the study is the diversity of the sample, which included medium and large dog breeds, as well as a similar number of males and females, which provides a strong foundation for evaluating the model’s performance across a range of anatomical variations.

To determine the ideal threshold for rejecting radiographs based on the Dys4Vet AOFA measurement model, we considered the acceptable asymmetry value as the mean of the minimal rotation group, in line with the clinical expectation of pelvic rotation up to 3 degrees stipulated in other studies [7,8], plus the model’s SEM. In addition to accounting for the natural variability reflected in the mean asymmetry (33.28 mm^2^), we included the measurement error (17.18 mm^2^) to avoid mistakenly rejecting radiographs that remain within an acceptable range. This approach led us to establish an acceptable pelvic asymmetry threshold of 50.46 mm^2^ for the Dys4Vet AOFA measurement model.

## 5. Conclusions

In conclusion, this study underscores the effectiveness of AOFA as a predictor of pelvic rotation and a reliable indicator of radiographic positioning quality. The substantial variation in AOFA values across different degrees of pelvic rotation supports its utility for automated measurement. The Dys4Vet AOFA model demonstrated proficient precision in segmenting the obturator foramina, establishing its reliability for clinical use. We are confident that the imprecisions identified in automated AOFA measurements can be easily addressed as more images are progressively incorporated into the training process.

## Figures and Tables

**Table 1:** Statistical descriptive analysis of the asymmetry in obturator foramen areas (mm^2^) by pelvic rotation group.

Rotation Group	*N*	Mean *	SD	Mean 95% CI		Min	Max
				Lower Bound	Upper Bound		
Minimal (0° to 2°)	71	33.28 ^a^	27.25	26.83	39.73	0.08	116.89
Moderate (3° to 4°)	41	54.73 ^b^	27.98	45.90	63.56	5.70	121.54
Marked (5° to 6°)	37	85.85 ^c^	41.31	72.08	99.62	21.36	163.96
Extreme (≥7°)	54	160.68 ^d^	64.21	143.15	178.20	59.10	400.26

* Means with different superscripts are statistically different (*p* < 0.01) in the post hoc Games–Howell test that followed Welch’s ANOVA.

## Data Availability

The data presented in this study are available from the corresponding author on request.
